# Clocks, Viruses, and Immunity: Lessons for the COVID-19 Pandemic

**DOI:** 10.1177/0748730420987669

**Published:** 2021-01-22

**Authors:** Shaon Sengupta, Louise Ince, Francesca Sartor, Helene Borrmann, Xiaodong Zhuang, Amruta Naik, Annie Curtis, Jane A. McKeating

**Affiliations:** * Perelman School of Medicine, University of Pennsylvania, Philadelphia, Pennsylvania, USA; † Children’s Hospital of Philadelphia, Philadelphia, Pennsylvania, USA; ‡ Institute of Translational Medicine and Therapeutics, University of Pennsylvania, Philadelphia, Pennsylvania, USA; § Chronobiology and Sleep Institute, University of Pennsylvania, Philadelphia, Pennsylvania, USA; || Departement de Pathologie et Immunologie, Geneva, Switzerland; ¶ Institute of Medical Psychology, Medical Faculty, Ludwig Maximilian University of Munich, Munich, Germany; # Nuffield Department of Medicine, University of Oxford, Oxford, UK; ** School of Pharmacy and Biomolecular Sciences, Tissue Engineering Research Group, Royal College of Surgeons in Ireland, Dublin, Ireland; †† Chinese Academy of Medical Sciences Oxford Institute, University of Oxford, Oxford, UK

**Keywords:** COVID-19, circadian rhythms, immunity, pandemic, chronotherapy, viruses, SARS-CoV-2

## Abstract

Circadian rhythms are evolutionarily conserved anticipatory systems that allow the host to prepare and respond to threats in its environment. This article summarizes a European Biological Rhythms Society (EBRS) workshop held in July 2020 to review current knowledge of the interplay between the circadian clock and viral infections to inform therapeutic strategies against SARS-CoV-2 and COVID-19. A large body of work supports the role of the circadian clock in regulating various aspects of viral replication, host responses, and associated pathogenesis. We review the evidence describing the multifaceted role of the circadian clock, spanning host susceptibility, antiviral mechanisms, and host resilience. Finally, we define the most pressing research questions and how our knowledge of chronobiology can inform key translational research priorities.

## Introduction

The COVID-19 pandemic, caused by the SARS-CoV-2 coronavirus, is a global health issue with more than 69 million diagnosed infections and 1.58 million fatalities to date (December 2020), bringing unprecedented challenges for public health and clinical medicine. Policies are being formulated and therapeutic strategies pursued at unimaginable speeds. If successful, these strategies will limit the inexorable spread of this disease across the globe. Those of us studying circadian biology have been trying to understand how we can exploit our knowledge of chronobiology to inform the design and evaluation of interventions against SARS-CoV-2. While studies probing the direct connection between SARS-CoV-2 and circadian rhythms are limited, it is well accepted that the circadian clock regulates the host immune response and drug pharmacokinetics (Ruben et al., 2019). To explore how our existing knowledge of the interplay between the circadian clock and viruses may be relevant to SARS-CoV-2, European Biological Rhythms Society (EBRS) convened a virtual workshop in July 2020. This article summarizes the findings and key action points that emerged from this multidisciplinary discussion on the interface of circadian biology and immunity. While there are many excellent reviews on circadian biology and immunity (Mavroudis et al., 2013; Scheiermann et al., 2013; Butler and Gibbs, 2020; Nosal et al., 2020; Shivshankar et al., 2020), our aim is to review the literature that is directly pertinent to SARS-CoV-2 and COVID-19 pathology. Furthermore, we speculate on the translational aspects of chronobiology that may impact this pandemic. Our hope is that this review will inform those engaged in research, public health policy decision, and clinical practice.

### The Biological Clock

**Figure 1.**
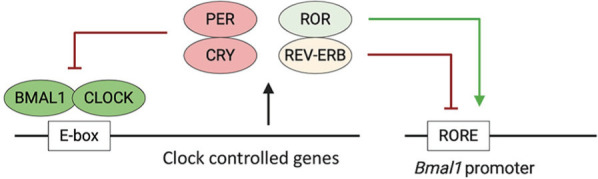
The molecular clock. Circadian rhythms are regulated by self-sustaining transcriptional/translational feedback loops. The transcription factors BMAL1:CLOCK bind to E-Boxes and activate the expression of clock genes, among others *Rev-erb*, *Ror*, *Per*, and *Cry* genes. PER:CRY directly inhibit BMAL1:CLOCK; REV-ERB inhibits and RORα activates *Bmal1* promoter activity. Color version is available online.

Circadian rhythms are an evolutionarily conserved biological system that serves to maintain organismal homeostasis. At the molecular level, the clock comprises core clock genes that activate or suppress downstream targets through rhythms in transcription, translational, and posttranslational mechanisms (Figure 1) (McDearmon et al., 2006; Ray et al., 2020). The molecular clock has several layers of redundancy built in to its organization. However, *Bmal1* is the only nonredundant gene whose sole deletion is sufficient to produce a phenotype of circadian arrhythmia, characterized by loss of rhythmicity in locomotor function and body temperature. Thus, the manipulation of *Bmal1* serves as a popular model for circadian studies. It is increasingly recognized that immune function is regulated in a rhythmic manner and is sensitive to the timing of pathogen exposure (Scheiermann et al., 2018; Diallo et al., 2020; Haspel et al., 2020). Since SARS-CoV-2 primarily replicates in the respiratory tract, a circadian-regulated system (Paschos and FitzGerald, 2010; Ince et al., 2018), diurnal rhythms are likely to have direct relevance to COVID-19 pathology. While most therapeutic strategies being tested are targeted to specific steps in the virus replicative life cycle or host immune response, the circadian system, given its integrative role in maintaining organismal homeostasis, promises many potential opportunities.

## Influence of Circadian Clock on Viral Infections

As obligatory intracellular parasites, viruses depend on the host cellular machinery to replicate. Since circadian pathways can regulate host gene expression, protein modification, and metabolism, it is not surprising that multiple steps in the viral life cycle are under circadian control (Borrmann et al., 2021). Viruses have either DNA or RNA genomes (Durmuş and Ülgen, 2017), and this can define whether circadian factors regulate viral transcription by directly binding to the viral genome or via indirect regulation of host genes. The outcome of most viral infections reflects the balance between viral proliferation and host antiviral immune responses.

One of the ways to investigate the circadian influence over a biological process is to conduct the experiment at different times of the day. Multiple studies have reported that the severity of viral infection is dependent on the circadian phase at which the host encounters the pathogen (reviewed in Borrmann et al., 2021). SARS-CoV-2 primarily targets the respiratory tract (Hou et al., 2020) and can result in pneumonia and acute respiratory distress syndrome especially in the elderly and in those with comorbidities (Guan et al., 2020). Recent studies reporting multiorgan involvement in severe COVID-19 disease (Gavriatopoulou et al., 2020), including the gastrointestinal tract (Trottein and Sokol, 2020) and central nervous system (Marshall, 2020), highlight the complexities of treating this disease. Since the mechanisms underlying the circadian control of virus infection and associated pathology are likely to be complex, reflecting sites of viral replication and pathogen-specific steps in their life cycle, we will review studies investigating the circadian regulation of respiratory viruses.

Ehlers et al. (2018) evaluated the effect of *Bmal1* deletion on the host response to Sendai virus, a pathogen used to simulate acute bronchiolitis associated with respiratory syncytial virus in humans. In mammals, the primary external time cue (zeitgeber, ZT) is the light/dark cycle that transduces signals through the retinohypothalamic tract to the suprachiasmatic nucleus (SCN) or “Master Clock” in the brain. Loss of *Bmal1* led to increased weight loss, elevated viral burden, and more severe lung pathology in mice infected between ZT4 and ZT8 (where ZT0 refers to the time of “lights-on”) compared to their wild-type littermates. Furthermore, following recovery, the lung showed signs of a post-viral pathology consisting of increased mucus metaplasia and greater airway reactivity. Under chronic jetlag conditions, progressive shifts in the light/dark cycles over a period of time cause a misalignment of various behaviors with endogenous circadian rhythms. Chronic jetlag was reported to phenocopy *Bmal1* deletion, and this was more pronounced in 12-month-old mice. An independent study by Sengupta et al. (2019) showed that mice infected with the murine-adapted H1N1 influenza type A virus (IAV) at ZT23 (dawn or just before lights go on) showed 3-fold increased survival relative to mice infected at ZT11 (dusk or just before lights go off). This time-of-day-specific effect was lost in mice where *Bmal1* was deleted in adulthood, demonstrating a role of the clock in providing a survival benefit to IAV infection. Interestingly, a club cell–specific *Bmal1* knockout (KO) showed the same effect as the global *Bmal1* KO, suggesting a primary role for this lung epithelial cell in mediating the circadian regulation of clock IAV. Z. Zhang et al. (2019) reported that club cell–specific *Bmal1* KO mice showed increased morbidity and persistent inflammation 11-21 days after IAV infection. These studies highlight the following salient points:

- The time of day at which the host is exposed to an infectious agent changes the outcome even weeks to months later. This relationship is likely to depend on the tropism of the viral pathogen and is affected by the age of the host.
- Overall, circadian disruption, whether through jetlag or genetic models, leads to a hyperinflammatory state with more severe outcomes following viral infection.
- The lung epithelium plays a crucial role in mediating the clock gating of lung injury.
- Several components of the immune system may contribute to the circadian regulation of lung inflammation.
- The effect of the circadian clock on pathogen replication is dependent on the virus type.

## Potential Influence of Circadian Rhythms ON SARS-CoV-2 Infection

**Figure 2.**
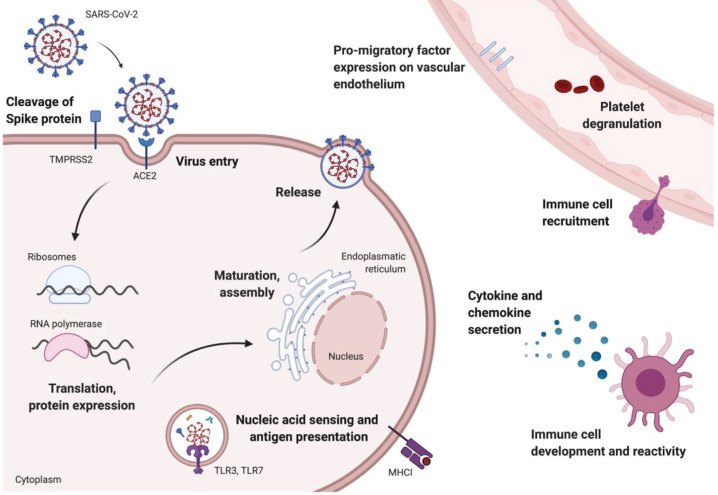
Potential circadian regulation of SARS-CoV-2 replication and host immune responses. Multiple steps of the viral life cycle involve circadian-regulated host pathways, which could affect SARS-CoV-2 replication. In addition, many aspects of the host immune response are circadian-regulated and may influence the severity of the inflammatory response to SARS-CoV-2 and the severity of COVID-19. Color version is available online.

The severity of COVID-19 pathology reflects a balance between SARS-CoV-2 replication and host immune responses, providing multiple points where the circadian clock and diurnal rhythms may regulate this disease (Lucas et al., 2020) (Figure 2).

### Viral Entry and Replication

SARS-CoV-2 infection is mediated by the viral-encoded Spike protein binding to human angiotensin-converting enzyme (ACE2), with transmembrane proteases (Hoffmann et al., 2020; Wan et al., 2020), serine 2 (TMPRSS2), and furin triggering fusion of the viral and cell membranes. There is limited evidence of oscillations in either ACE2 or TMPRSS2 in human airways; however, a very modest oscillation in *Ace2* messenger RNA (mRNA) was reported in rat heart, with a peak at the start of the rest phase (Herichova et al., 2013). Analyzing published diurnal/circadian transcript data sets shows limited evidence for a rhythmic expression of *Ace2* in the lung of humans (Anafi et al., 2017), baboons (Mure et al., 2018), or mice (Z. Zhang et al., 2019). However, there was a modest increase in *Ace2* transcript (1.3-fold of wild-type levels) in *Bmal1* KO bronchial epithelial cells (Z. Zhang et al., 2019), suggesting that BMAL1 or other clock components may regulate *Ace2* expression in these cells. The human *Ace2* promoter (Eukaryotic Promoter Database, 2020) encodes potential binding sites for BMAL1 and CLOCK (Dreos et al., 2017), consistent with a role of these circadian components to regulate ACE2 expression and susceptibility to SARS-CoV-2 infection. *Tmprss2* was not rhythmically expressed in the murine and baboon mRNA data sets but showed some evidence of cycling in the human lung (Anafi et al., 2017). In mouse liver, an intronic region was found to bind to both CREB-binding protein (CBP) and the negative arm of the core clockwork (CRY2, CRY1, PER1, PER2), raising the possibility of a clock-regulated enhancer site (Koike et al., 2012). These studies show the potential for ACE2 and TMPRSS2 to be regulated by the circadian clock, but specific assessment of these proteins and their function as receptors to allow SARS-CoV-2 infection in physiologically relevant cell populations such as the vascular endothelium and pulmonary epithelium has yet to be undertaken.

Once inside the cell, coronaviruses (as positive-sense single-stranded RNA viruses) utilize host ribosomes to translate viral RNA. Ribosomal rhythms (Jouffe et al., 2013; Jang et al., 2015; Sinturel et al., 2017) may alter SARS-CoV-2 replication capacity by providing an oscillating source of host machinery (Figure 2). Previous studies showed that murid herpesvirus 4 (muHV-4) replication was enhanced when mice were infected at the onset of the rest phase, coinciding with the time of peak expression of proteins regulating RNA biosynthesis (Edgar et al., 2016). Furthermore, infection with either muHV-4 or IAV was augmented in *Bmal1* KO cells, which also showed an increased abundance of proteins regulating translation, protein folding, and intracellular vesicle trafficking. The influence of biological rhythms on SARS-CoV-2 virus entry and replication is an important area for investigation. Such information could identify peak times for risk of infection and may focus our attention on clock-disrupted groups such as shift workers or those with circadian sleep disorders who may be at higher risk of infection.

## Host Immune Responses and COVID-19 Pathology

The second, and perhaps more significant, rhythmic feature regulating COVID-19 pathology is the type of host immune response that can define the severity of inflammation. In all cases, more severe pathology was associated with a proinflammatory cytokine signature (Blanco-Melo et al., 2020) and an influx of circulating immune cells such as neutrophils, natural killer cells, and monocytes. Under steady state conditions, oscillations occur in circulating immune cell numbers as cells migrate through the bloodstream into tissues and recirculate through lymphatics. Peripheral leukocytes peak during the rest phase in both mice and humans (Suzuki et al., 2016; Druzd et al., 2017; He et al., 2018), and this rhythmic phenotype is regulated by the cellular pacemakers of both leukocytes and the vascular microenvironment (Jang et al., 2015; Zhao et al., 2017), along with adrenergic and glucocorticoid signaling (Scheiermann et al., 2012; Suzuki et al., 2016; Shimba et al., 2018). Furthermore, diurnal fluctuations in the expression of albumin/fibrinogen levels in human nasal samples (Greiff et al., 1996) suggest that components of the mucosal barrier may also be clock-controlled. Tissue-specific diurnal changes occur in the expression of promigratory factors on the vascular endothelium (He et al., 2018), leading to rhythms in migratory drive under homeostatic conditions. In an inflammatory scenario, there is a predisposition for enhanced recruitment of immune cells to infected tissue at particular times of the day, although this has not yet been directly studied in a COVID-19 model.

In addition to diurnal changes in immune cell numbers, oscillations in cell reactivity are a significant feature determining the inflammatory response. One of the most well-characterized diurnal patterns of immune cell responsiveness is that of the neutrophils. Although these cells are relatively short-lived, they exhibit an aging process whereby cells circulating in the active phase are “fresh,” whereas those in the rest phase display an “aged” phenotype and are cleared back to the bone marrow (Adrover et al., 2019). Rather than simply being a measure of how long cells have been in the circulation, these distinctions have functional relevance for pathophysiology as mice with constitutively aged neutrophils are resistant to infection but experience enhanced vascular injury and thrombosis. Similar oscillations were reported in the reactivity of circulating monocytes (Nguyen et al., 2013), as well as in the aforementioned tissue-resident macrophages and lung epithelium, making it highly likely that oscillations occur in the innate immune response to SARS-CoV-2 infection. As discussed earlier, in a mouse model of IAV infection, time of exposure was a significant determinant of survival that was dependent on the inflammatory responses required to clear infection, with infection at ZT11 leading to more severe pathology (Sengupta et al., 2019). In models utilizing PolyI:C (a TLR3 ligand), greater tissue damage was observed after administration during the transition from the active to the rest phase (Gibbs et al., 2014; Ince et al., 2019; Sengupta et al., 2019).

As immune responses transition from the early innate arm to adaptive long-term protection, the focus shifts toward T- and B-cell reactivity in secondary lymphoid organs. Although adaptive responses occur on a longer time frame than the acute inflammatory phase, the timing of pathogen exposure remains a key determinant. Mice infected during the rest phase exhibit increased cellularity in their draining lymph nodes and enhanced autoimmune pathology even weeks after exposure (Druzd et al., 2017; Sutton et al., 2017), potentially driven by rhythms in T-cell proliferation capacity (Fortier et al., 2011; Nobis et al., 2019). Furthermore, human CD4^+^ T-cells showed a circadian variation in activation based on the time of stimulation by phorbol myristate acetate (PMA)/ionomycin or phytohemagglutinin (PHA) (Bollinger et al., 2011; Cuesta et al., 2016). The transcriptome of CD8^+^ T-cells showed circadian oscillations in various effector molecular pathways (Nobis et al., 2019). Much less is known about the temporal regulation of B-cell responsiveness and antibody production, but it is likely that this would follow a similar pattern given their dependence on T-cell support.

Circadian rhythms governing host immune response have direct implications for cell proliferation, recruitment, reactivity, and development of inflammatory sequalae. It is crucial that we understand whether these inflammatory rhythms change in an active infection scenario and how this can be translated into therapeutic strategies. For example, appropriate timing of treatments and preventive measures such as vaccinations to target the host’s endogenous rhythms may boost the efficacy of these interventions. In fact, preliminary evidence suggests that providing antiviral therapies in the morning versus evening may lead to increased efficacy (De Giorgi et al., 2020). Another facet is that of circadian disruption, ubiquitous with our current lifestyle. In numerous models of viral infection as well as aseptic inflammation, the disruption of the clock incurred by the loss of *Bmal1* in key cell types results in increased disease severity (Scheiermann et al., 2012; Gibbs et al., 2014; Sutton et al., 2017; Early et al., 2018; Ehlers et al., 2018; Pariollaud et al., 2018; Sengupta et al., 2019; Z. Zhang et al., 2019). Given the tightrope walk of generating an effective but not overly destructive immune response, it would not be surprising that circadian disruption may bias the immune response to exaggerated inflammatory response and hence severity of COVID-19. Excessive inflammation originating in the lung may propagate systemically via persistent elevated cytokine release (cytokine storm) that overwhelms the body and causes multiorgan damage.

### SARS-CoV-2 Perturbation of Host Circadian Rhythms

Given the close interaction of the clock and viral pathogens, another intriguing possibility is that the SARS-CoV-2 infection could directly or indirectly affect clock function in infected cells and cause desynchrony with the surrounding tissue. IAV infection altered the timing of *Bmal1*, *Clock*, and *Rev-erbβ* peak expression in the lungs of infected mice and reduced the amplitude of *Per2* expression in lung tissue explants. However, the underlying mechanisms are not defined (Sundar et al., 2015). SARS-CoV-2 infection disrupts several physiological processes that include diverse metabolic pathways, macrophage function, cellular oxidant detoxification, and platelet degranulation (Bojkova et al., 2020). Many of these processes are circadian-regulated, consistent with SARS-CoV-2 infection inducing a perturbation in circadian signaling.

Oxygen has been identified as a cue for entraining molecular clocks in several vertebrate animal models (Chilov et al., 2001; Ghorbel et al., 2003). The cellular response to low oxygen is regulated by hypoxia-inducible factors (HIFs), members of the basic helix-loop-helix-PAS family of transcription factors that includes BMAL1. HIF and BMAL1 share homologies in their dimerization domains and DNA-binding motifs, enabling cross-talk between the circadian and hypoxia signaling pathways (O’Connell et al., 2020; Peek, 2020). Tissue oxygenation and oxygen consumption show daily rhythms in mice (Adamovich et al., 2017; Adamovich et al., 2019), and in vitro studies show that cellular clocks are synchronized in an HIF-dependent manner (Adamovich et al., 2019). Hypoxia can alter the oscillation of physiological variables such as body temperature, metabolic rate, cortisol, and melatonin release in humans (Bosco et al., 2003; Coste et al., 2004). SARS-CoV-2 infection of monocytes was reported to stabilize HIFs that induced glycolytic metabolism and inflammatory responses (Codo et al., 2020), providing a potential mechanism for SARS-CoV-2 to perturb circadian gene expression. COVID-19 pneumonia is atypical and associated with hypoxemia (Gattinoni et al., 2020; Radovanovic et al., 2020), and this could have profound effects on local and systemic circadian rhythms. Many patients would have received oxygen, and it is interesting to consider the potential effects of this therapy on clock-regulated pathways and disease outcomes. Importantly, several reports show that supplemental oxygen given to premature infants associates with a risk of lung dysfunction and susceptibility to respiratory infections in adult life (Buczynski et al., 2013; Yee et al., 2013; Kumar et al., 2018).

## Translational Opportunities for COVID-19

**Figure 3.**
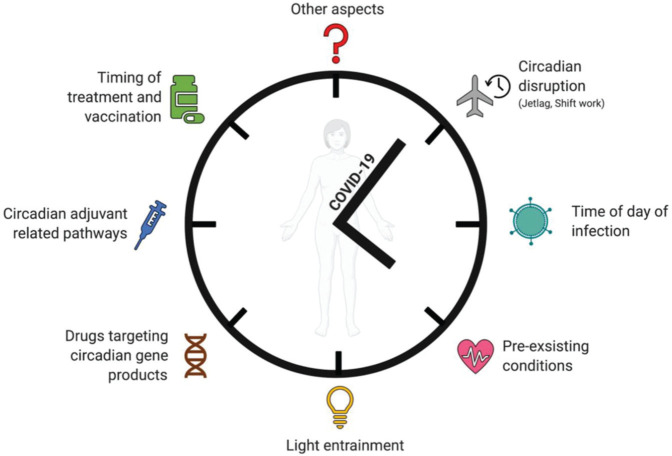
Translational effects of circadian rhythm on COVID-19. Circadian rhythms affect disease outcomes, which may impact COVID-19 severity.

Circadian clocks have important implications in the pharmacokinetics and pharmacodynamics of drug responses (Ruben et al., 2019). Rhythmic alterations in drug absorption, distribution, metabolism, and excretion in the expression of target receptors influence the bioavailability and efficacy of a drug. Therefore, strategic options include (1) “clocking the drug”—identifying the most optimal time to administer the drug to achieve the optimal balance of efficacy versus toxicity, (2) “drugging the clock”—pharmacological targeting of circadian components to modulate disease outcomes, or (3) “training the clock”—incorporating lifestyle interventions such as time-restricted feeding or lighting exposure to improve circadian function (Sulli et al., 2018) (Figure 3). Remarkably, studying mice transcriptomic data showed that 56 of the 100 best-selling drugs target the products of circadian-regulated genes (R. Zhang et al., 2014). Similarly, a human transcriptomic study identified that nearly half of the protein-coding genes were cycling in 13 tissues analyzed. Interestingly, 1000 of these cycling genes encode proteins that drugs target or that are involved in drug metabolism or transport (Ruben et al., 2018). Hundreds of genes in the human epidermis are rhythmically expressed, which helped to identify diagnostic biomarkers to inform the circadian phase of a subject from a single sample (G. Wu et al., 2018). We propose the following areas where circadian biology offers the potential to impact the treatment of COVID-19.

## Circadian Biology in Vaccine Development and Testing

There is increasing evidence that the time of day of vaccine administration influences the host response. From a COVID-19 perspective, this is perhaps the most promising and imminently actionable item to intersect ongoing studies with chronobiology. At the time of proof-reading this article (2 December 2020), 32 vaccine candidates were being tested in clinical trials and 3 successful phase 3 trials that have been completed are undergoing final review prior to approval for use (New York Times Coronavirus Tracker, 2020; Ramasamy et al., 2020). Now, at least two major groups of vaccines have been or are close to being approved with good efficacy against SARS-CoV-2 (Polack et al., 2020; Voysey et al., 2020). To the best of our knowledge, the current SARS-CoV-2 vaccine studies are not designed to assess the time of day of vaccination; however, few studies that have attempted this report exciting results. A randomized trial of 276 adults aged above 65 years demonstrated that morning vaccination (9-11 am) for influenza resulted in a greater antibody response compared to those vaccinated in the afternoon (3-5 pm) (Long et al., 2016). An independent study design showed that the time of immunization influenced the induction of trained immunity (de Bree et al., 2020). In a cohort of 302 subjects, those inoculated with the Bacillus Calmette-Guerin (BCG) vaccine between 8 and 9 am showed stronger trained immunity than those vaccinated between noon and 1 pm (de Bree et al., 2020). It is also interesting to note that regular sleep after hepatitis A vaccination enhanced the antibody response (Lange et al., 2003; Lange et al., 2011). Although there are numerous differences between these studies, it is intriguing to note that both BCG, a live attenuated vaccine, and influenza, a subunit vaccine, showed enhanced efficacy in the morning. In addition to these two clinical studies, there have been more mechanistic work in animals (Silver et al., 2012; Nobis et al., 2019), which provides a sound biological basis for the circadian influence on vaccine efficacy.

The time frame for vaccine development can take up to 15 years; however, the urgency for a SARS-CoV-2 vaccine is compressing this time scale into months. There are opportunities here that are unparalleled in any other immunization campaign. Given the recent emergence of SARS-CoV-2, assessing the impact of time of day of administration on the protection generated is unlikely to be confounded by previous vaccinations or exposures. Even if optimizing the time of day of vaccination only provides a small increase in efficacy, this could have a major impact on the public health level since billions of individuals will be receiving a SARS-CoV-2 vaccine. Parallel studies could dissect the molecular mechanisms underpinning circadian-driven effects. Thus, time-stamping SARS-CoV-2 vaccine administration is biologically justified and is a relatively simple task that could yield critical information for the future success of the vaccines targeting this global pandemic.

## Circadian Biology in Drug Development and Testing

“Clocking the drug” or a chronotherapeutic approach is also relevant to the experimental treatments under investigation for COVID-19. Approximately 80% of patients recover from infection without the need for treatment (Z. Wu and McGoogan, 2020). However, for those who are significantly affected by the virus, treatment options fall into 2 categories: drugs to suppress viral replication and therapies to dampen the overactive host immune response and cytokine storm that drives severe lung and systemic pathology. The major pharmaceuticals being evaluated include the antiviral agent remdesivir, interferon B-1a, monoclonal antibodies against interleukin 6 (IL-6) and IL-4, and dexamethasone. Dexamethasone (Villar et al., 2020) and remdesivir (Beigel et al., 2020) have shown efficacy in randomized controlled trials. Interestingly, administration of prednisolone, a glucocorticoid-like dexamethasone, showed a time-of-day-dependent effect in alleviating rheumatoid arthritis symptoms (Buttgereit et al., 2008). Similarly, drugs that are known to reduce clinical symptoms in COVID-19 preclinical models such as ritonavir are sensitive to time of administration (De Giorgi et al., 2020); however, this has not been assessed for remdesivir. Generally, drugs with short half-lives (6 h or less) that include prednisolone are more sensitive to time of day of administration. In contrast, remdesivir has a significantly longer half-life (>24 h), and thus chronotherapy may not be beneficial. Although the most recent reports showed minimal efficacy of remdesivir in a larger cohort of patients (WHO Solidarity Trial Consortium et al., 2020), the circadian principles guiding the administration hold true for any drug with short half-life. As more drugs are evaluated for the treatment of COVID-19, it will be important to determine whether the drug target displays rhythmicity and/or to ascertain whether circadian features exist in the pharmacokinetic and dynamic properties of the drug.

Over the last decade, a number of compounds that directly target core clock proteins or proteins within ancillary loops have been developed (Ercolani et al., 2015). These compounds include agonists of REV-ERBs (Everett and Lazar, 2014; Wang et al., 2020) and receptor-related orphan receptors (RORs) (Solt and Burris, 2012), compounds that stabilize the cryptochrome proteins (Hirota et al., 2012) and inhibitors of casein kinase (CK) (Meng et al., 2010). Some of these clock modulators display antiviral activity, with recent reports of REV-ERBα agonists inhibiting both hepatitis C virus and HIV replication (Zhuang et al., 2019; Borrmann et al., 2020). REV-ERBα agonists also impact the host immune response by suppressing IL-6 (Gibbs et al., 2012) and may represent a “two-pronged” approach to reduce viral replication and adverse host immune responses. The CK2 inhibitor silmitasertib is being evaluated as an anticancer agent (Silva-Pavez et al., 2019) and was recently shown to inhibit SARS-CoV-2 replication by reducing filopodial protrusions of budding viral particles (Bouhaddou et al., 2020). Whether the role of CK2 (Silva-Pavez et al., 2019) as a clock protein is relevant for its antiviral mechanism is unknown. Looking forward, we should consider how to best exploit and manipulate the circadian clock to improve new therapies for the treatment of COVID-19.

## Realizing The Impact of Circadian Biology on COVID-19 Epidemiology

Both epidemiological (Pan et al., 2011; Vyas et al., 2012; Wegrzyn et al., 2017) and molecular evidence (Kervezee et al., 2018) show that night shift workers have circadian disruption and are at an increased risk of developing chronic inflammatory diseases. Mice subjected to a shift work regimen display a highly overactive dysregulated inflammatory response (Castanon-Cervantes et al., 2010). Identifying shift work as a risk factor for acquiring SARS-CoV-2 infection or developing a more severe disease would be informative for public health policy, especially as many of the health care professionals working on the front line for this pandemic are shift workers. Furthermore, time-of-day dependency may have consequences in terms of the likelihood of infection, host response to the infection, virus shedding and transmission, and subsequent disease severity from the infection (Figure 3). Understanding the circadian impact on SARS-CoV-2 epidemiology would allow us to make more informed decisions in terms of gatherings, be it for work, school, or social settings. Contact tracing data which are now being collected across the globe will answer the question of whether there is an association between the time of day of infection and severity of COVID-19 disease. This would be a significant step in curbing the spread of the virus among essential frontline staff, where risk-stratifying by time of day could optimize other measures of limiting spread. In conclusion, there are multiple steps during the viral infection and host response and recovery where the circadian clock and SARS-CoV-2 interact, and this forms a template for future research. While we continue to learn more about this pathogen, it is imperative that we build into our research studies the ability to probe for the effect of time of day of intervention in all our analyses.
